# Herpes zoster (HZ) vaccine coverage and confidence in Italy: a Nationwide cross-sectional study, the OBVIOUS project

**DOI:** 10.1186/s12879-024-09344-7

**Published:** 2024-04-24

**Authors:** Aurelia Salussolia, Angelo Capodici, Francesca Scognamiglio, Giusy La Fauci, Giorgia Soldà, Marco Montalti, Zeno Di Valerio, Maria Pia Fantini, Anna Odone, Claudio Costantino, Heidi J. Larson, Julie Leask, Jacopo Lenzi, Davide Gori, on behalf of the OBVIOUS board, Angelo Capodici, Michele Conversano, Claudio Costantino, Mirko Degli Esposti, Zeno Di Valerio, Maria Pia Fantini, Davide Gori, Andrea Grignolio, Giusy La Fauci, Heidi J. Larson, Julie Leask, Jacopo Lenzi, Marco Montalti, Anna Odone, Daniel Remondini, Francesca Scognamiglio, Aurelia Salussolia, Giorgia Soldà, Federico Toth, Francesco Vitale

**Affiliations:** 1 Department of Biomedical and Neuromotor Science, Alma Mater Studiorum - University of Bologna, Bologna, Italy; 2 Department of Public Health, Experimental and Forensic Medicine, University of Pavia, Pavia, Italy; 3 Department of Health Promotion Sciences, Maternal and Infant Care, Internal Medicine and Excellence Specialties “G. D’Alessandro” - University of Palermo, Palermo, Italy; 4 Infectious Disease Epidemiology, London School of Hygiene & Tropical Medicine Institute of Health Metrics & Evaluation, University of Washington, Seattle, USA; 5 Susan Wakil School of Nursing and Midwifery, Faculty of Medicine and Health, University of Sydney, Sydney, NSW Australia; 6 Sydney Infectious Diseases Institute, Westmead, NSW Australia

**Keywords:** Herpes zoster, Vaccine, Uptake, Hesitancy, Confidence, Safety perception

## Abstract

**Background:**

Herpes Zoster is an age dependent disease and as such it represents a problem in the Italian social context, where the demographic curve is characterized by an overrepresentation of the elderly population. Vaccines against Herpes Zoster are available, safe and effective, however coverage remains sub-optimal. This study was therefore conducted to examine the variations in Herpes Zoster vaccine uptake and confidence across different regions in Italy.

**Methods:**

This study utilized a cross-sectional computer-assisted web interview (CAWI) methodology. The survey was conducted by Dynata, an online panel provider, and involved 10,000 respondents recruited in Italy between April 11 and May 29, 2022. The sample was stratified based on geographic region, gender, and age group. Data management adhered to European Union data protection regulations, and the survey covered demographics, living conditions, and vaccination against herpes zoster (HZ), following the BeSD framework.

**Results:**

The findings indicate regional disparities in herpes zoster vaccine uptake across Italy. Notably, the Islands region exhibits a particularly low vaccination rate (2.9%), highlighting the need for targeted interventions. The multivariate regression analysis showed that sociodemographic factors, limited access to healthcare services, and inadequate awareness of vaccine eligibility contribute to the lower uptake observed in this region.

**Conclusion:**

In conclusion, this research emphasizes regional disparities in herpes zoster (HZ) vaccination uptake in Italy. Demographic, socioeconomic, and geographic factors impact individuals’ willingness to receive the vaccine. The study highlights the importance of awareness of vaccine eligibility and accessible vaccination facilities in increasing uptake rates.

**Supplementary Information:**

The online version contains supplementary material available at 10.1186/s12879-024-09344-7.

## Introduction

Herpes Zoster (HZ), also known as shingles, is an acute disease caused by the reactivation of the latent form of Varicella Zoster Virus (VZV) in dorsal root ganglia [1, 2]; clinically, it presents a vesicular rash within a dermatomal distribution, accompanied by malaise and other possible complications. The most prevalent long-term complication is Post-Herpetic Neuralgia (PHN), affecting approximately 10 to 20% of individuals diagnosed with HZ [1–3].

As an age-dependent disease, HZ predominantly affects individuals over 50 years old. Ageing causes a decline in VZV-specific lymphocytes, with 20% of subjects aged between 55 and 65 years having an undetectable cell-mediated response against VZV [1, 4].

In the context of Italy’s ageing population, HZ and its associated complications may emerge as increasingly prominent public health challenges. Data regarding the burden of the disease and its economic impact in Italy mainly come from a retrospective investigation conducted by Gialloreti et al., who estimated an incidence of 6.3 cases per 1000 person-years [1], and another study by Alicino et al. [5], who translated this value into a total economic burden of 41.2 million euros per year.

Considering these factors, the vaccination of the older population to mitigate the impact of HZ, is of paramount importance. Presently, there are two vaccines against HZ available: the live attenuated vaccine, and the adjuvanted recombinant vaccine [6].

In Italy, HZ vaccination is offered freely to individuals aged 65 or older, or those aged 50 and above presenting risk factors such as diabetes mellitus, cardiovascular diseases, chronic pulmonary diseases, or individuals undergoing immunosuppressive therapy. Prior to 2021, only the live attenuated vaccine was available in Italy, capable of reducing approximately 65% of PHN cases and about 50% of all HZ clinical cases [7].

Taking into account the aforementioned evidence, and considering HZ vaccines appear to be both effective and safe [8], thereby serving as a crucial public health measure to alleviate the burden of disease. Hence, it is key to encourage the HZ vaccine uptake among the target population, mitigating the vaccine hesitancy pertaining to this immunisation. Vaccine hesitancy was defined by the WHO as the reluctance or refusal to vaccinate despite the availability of vaccines, marking as underlying causes complacency, inconvenience, and lack of confidence [9]. In order to understand potential drivers of vaccine uptake and develop targeted and cost-effective interventions, it was deemed necessary to assess and characterise the confidence toward this specific vaccination in the Italian context.

Unfortunately, to our knowledge, vaccine uptake and confidence regarding HZ vaccines has not been studied yet in Italy, and neither in large European populations. Alas, we can only refer to the international scenario to better grasp how this vaccine is perceived by different populations.

A study by Lu et al. [10] found low willingness to vaccinate against shingles in a Chinese population sample aged 50-69 years., reporting how the willingness to receive the vaccine increased if the vaccination costs were covered by insurance and not directly paid by the subjects.

Vaccine cost was deemed as an important factor in diminishing vaccine uptake in other studies, which found other drivers, like inadequate vaccine supplies, perceived barriers to the access, as well as previous episodes of shingles as important hesitancy factors [11, 12].

This study aims to describe not only the vaccine uptake in the target population for HZ vaccine, but also to analyse possible determinants of vaccine hesitancy, to enable a better understanding of the phenomenon and provide resourceful data to counteract it.

## Methods

### Study design and data collection

This study was conducted using a cross-sectional computer-assisted web interview (CAWI). The survey was conducted by Dynata, a professional online panel provider, between April 11 and May 29, 2022, and a national sample of 10,000 respondents was recruited. The sample was defined using a stratified sampling based on proportionate allocation by first-level NUTS (Nomenclature of Territorial Units for Statistics) statistical region of residence (Northwest, Northeast, Center, South, and Islands), gender, and age group (18–24, 25–34, 35–44, 45–54, 55–64, and ≥ 65 years). Post-stratification confirmed that non-response to the survey in some strata of Italy’s adult target population had no substantial effect on the study estimates [13]. For this reason, adjustment of sampling weights was deemed as unnecessary to be performed on the target subsample of respondents for zoster vaccination (*n* = 1890). The data management was performed in accordance with the General Data Protection Regulation of the European Union. The survey experiment followed all requirements under Italian regulations.

### Data

The seven-sections OBVIOUS questionnaire was designed to be completed in ~ 10 minutes and aimed at investigating: demographics and living conditions, data on vaccination against HZ (the focus of this work), pneumococcus, HZ virus, rotavirus and human papillomavirus, political orientation, and attitudes toward SARS-CoV-2 vaccination, science, and alternative medicines.

The HZ section was administered to the populations that the Italian Ministry of Health guidelines identifies as vaccination targets, i.e. people aged ≥65 years and people aged ≥50 years with underlying diseases.

### Variables & Statistical Analysis

The questionnaire was developed based on the BeSD (Behavioural and Social Drivers of Vaccination) report on vaccine uptake [14]. The first section (sociodemographic) included18 questions: gender; date of birth; educational level; region of residence; occupation; weight; height; living conditions; ability to meet one’s needs with current income; being a parent; birth date, age and sex of the youngest child; presence of physical or mental impairment; presence of diabetes/respiratory/cardiovascular disease; where the person got the most vaccinations; where the person would have preferred to get most of the vaccinations; family and friends’ view on vaccinations.

Following this sociodemographic section, the questionnaire investigated 6 variables specific to the HZ vaccination, such as: if the respondent received the HZ vaccine; if not, whether they intended to; vaccine’s perceived safety; perceived concern about getting the disease; knowledge about the right to get the shot; and the perceived accessibility of the vaccination.

An English version of the survey questions can be found in the Supplementary Material - Questionnaire.

All variables were summarised as counts and percentages, and were stratified by first-level NUTS statistical region of residence, gender, and target group based on age and/or clinical status (ages ≥65 years vs. ages ≥50 years in conjunction with diabetes, pneumopathy or cardiopathy). Data were visualised with the aid of square charts and thematic maps with pie charts. Square charts, also called waffle charts, are a form of pie charts that use 10 × 10 grids instead of circles to represent percentages.

Multivariable multinomial logistic regression analysis was carried out to examine the drivers (determinants) of vaccine hesitancy, which was considered as a three-category nominal outcome (“I did get the vaccine” vs. “I did not get the vaccine, but I would” vs. “I do not want to get vaccinated”). In keeping with the increasing vaccination model proposed by the BeSD Expert Working Group [14], the covariates included in the regression model as potential drivers of vaccine hesitancy were the following: thoughts and beliefs about HZ infection and vaccination (perceived worry and safety concerns), social processes (friends and family’s views on vaccination, gender), and practical issues (awareness of having higher priority for vaccination, and perceived ease of access to healthcare to get the vaccine). Relevant sociodemographic determinants were also considered (age group, statistical region of residence, place of residence, degree of urbanisation, and educational attainment), as well as clinical factors that lead to a higher priority for vaccination (diabetes, pneumopathy, and cardiopathy). The effect of covariates was assessed by examining the marginal effect of changing their values on the average predicted probability of observing each outcome. The marginal effect was computed as a discrete difference in probabilities (Δ), with 95% confidence intervals (CIs) obtained with the delta method. Covariate categories occurring in < 5% of the sample were combined with adjacent lower or upper classes to improve the stability and efficiency of regression estimates. The Small–Hsiao test of independence of irrelevant alternatives (IIA) did not indicate the need for alternative model specifications in which binary logit coefficients do not converge in probability to the same values as the multinomial logit coefficients, such as the nested logit model. Lastly, in order to check for the presence of moderators, that is, covariates Z that change the effect of other independent variables X on vaccine hesitancy, we included pairwise interaction terms Z × X in the model one at a time, and tested their statistical significance with the likelihood-ratio (LR) test. To control for type I error related to multiple testing, the significance level for interactions was set at 0.01.

All analyses were conducted using Stata software, version 17 (StataCorp. 2021. Stata Statistical Software: Release 17. College Station, TX: StataCorp LP). No multi-collinearity issues were found in regression analysis, that is, the variance inflation factor was < 5 and the condition index was < 10 for each covariate.

## Results

The sociodemographic characteristics of the respondents, overall and by geographical region, are summarised in Table 1. Among the 10,000 respondents, 1890 were in target for the HZ section and answered the questions regarding the vaccination; of them, 80 reported that they did not remember whether they had been vaccinated and thus were not considered for the analysis. Males represented 56.5% of the sample, while females 43.4%; the majority of the respondents were aged ≥65 years (76.4%), living in towns or suburbs (47.4%), and living in a couple (72.0%); in addition, 56.8% reported some financial difficulties in providing for themselves.

**Table 1:** Sociodemographic characteristics of the study sample, overall and by NUTS statistical region

Characteristic	Italy	Northwestern Italy	Northeastern Italy	Central Italy	Southern Italy	Insular Italy
	(*n* = 1810)	(*n* = 553)	(*n* = 361)	(*n* = 328)	(*n* = 396)	(*n* = 172)
Gender						
Male	1022 (56.5%)	298 (53.9%)	228 (63.2%)	190 (57.9%)	220 (55.6%)	86 (50.0%)
Female	785 (43.4%)	255 (46.1%)	133 (36.8%)	138 (42.1%)	173 (43.7%)	86 (50.0%)
Non-binary	3 (0.2%)	0 (0.0%)	0 (0.0%)	0 (0.0%)	3 (0.8%)	0 (0.0%)
Age group, y						
50–64	428 (23.6%)	118 (21.3%)	93 (25.8%)	76 (23.2%)	96 (24.3%)	45 (26.2%)
≥ 65	1382 (76.4%)	435 (78.7%)	268 (74.2%)	252 (76.8%)	300 (75.8%)	127 (73.8%)
Place of residence degree of urbanisation^a^						
City	698 (38.6%)	232 (42.0%)	104 (28.8%)	123 (37.5%)	189 (47.7%)	50 (29.1%)
Town or suburb	858 (47.4%)	264 (47.7%)	161 (44.6%)	155 (47.3%)	172 (43.4%)	106 (61.6%)
Rural area	254 (14.0%)	57 (10.3%)	96 (26.6%)	50 (15.2%)	35 (8.8%)	16 (9.3%)
Educational attainment						
Less than high school diploma	323 (17.8%)	107 (19.3%)	68 (18.8%)	50 (15.2%)	59 (14.9%)	39 (22.7%)
High school diploma	1051 (58.1%)	348 (62.9%)	174 (48.2%)	199 (60.7%)	232 (58.6%)	98 (57.0%)
Academic degree	288 (15.9%)	72 (13.0%)	57 (15.8%)	59 (18.0%)	71 (17.9%)	29 (16.9%)
Post-graduate degree	148 (8.2%)	26 (4.7%)	62 (17.2%)	20 (6.1%)	34 (8.6%)	6 (3.5%)
Occupation						
Teacher	77 (4.3%)	7 (1.3%)	38 (10.5%)	5 (1.5%)	22 (5.6%)	5 (2.9%)
Medical doctor	18 (1.0%)	6 (1.1%)	6 (1.7%)	4 (1.2%)	2 (0.5%)	0 (0.0%)
Healthcare worker (excl. Medical doctor)	18 (1.0%)	3 (0.5%)	7 (1.9%)	2 (0.6%)	4 (1.0%)	2 (1.2%)
Law enforcement member	15 (0.8%)	2 (0.4%)	9 (2.5%)	4 (1.2%)	0 (0.0%)	0 (0.0%)
Student	1 (0.1%)	0 (0.0%)	1 (0.3%)	0 (0.0%)	0 (0.0%)	0 (0.0%)
Other occupation	358 (19.8%)	112 (20.3%)	67 (18.6%)	68 (20.7%)	79 (19.9%)	32 (18.6%)
Unemployed	171 (9.4%)	46 (8.3%)	14 (3.9%)	30 (9.1%)	49 (12.4%)	32 (18.6%)
Retired	1152 (63.6%)	377 (68.2%)	219 (60.7%)	215 (65.5%)	240 (60.6%)	101 (58.7%)
Household composition						
Alone	303 (16.7%)	119 (21.5%)	61 (16.9%)	54 (16.5%)	47 (11.9%)	22 (12.8%)
Couple	1304 (72.0%)	379 (68.5%)	266 (73.7%)	236 (72.0%)	301 (76.0%)	122 (70.9%)
With parents/family	94 (5.2%)	24 (4.3%)	16 (4.4%)	13 (4.0%)	26 (6.6%)	15 (8.7%)
Other	109 (6.0%)	31 (5.6%)	18 (5.0%)	25 (7.6%)	22 (5.6%)	13 (7.6%)
Able to pay for things needed in life						
With great difficulty	263 (14.5%)	62 (11.2%)	32 (8.9%)	58 (17.7%)	79 (19.9%)	32 (18.6%)
With some difficulty	765 (42.3%)	217 (39.2%)	128 (35.5%)	158 (48.2%)	169 (42.7%)	93 (54.1%)
Quite easily	657 (36.3%)	237 (42.9%)	142 (39.3%)	101 (30.8%)	136 (34.3%)	41 (23.8%)
Easily	125 (6.9%)	37 (6.7%)	59 (16.3%)	11 (3.4%)	12 (3.0%)	6 (3.5%)

^a^According to the Eurostat Degree of Urbanization (DEGURBA) classification system

Northwestern Italy includes the regions of Piedmont, Aosta Valley, Lombardy, and Liguria; Northeastern Italy includes the regions of Trentino-South Tyrol, Veneto, Friuli-Venezia Giulia, and Emilia-Romagna; Central Italy includes the regions of Tuscany, Umbria, Marche, and Lazio; Southern Italy includes the regions of Abruzzo, Molise, Campania, Apulia, Basilicata, and Calabria; Insular Italy includes the regions of Sicily and Sardinia

*NUTS* Nomenclature of Territorial Units for Statistics

Considering the clinical characteristics of the participants, summarised in Table 2, 12.0% reported problems with daily living tasks due to physical or mental impairment, 24.4% reported suffering from diabetes, 19.0% from cardiovascular diseases and 12.7% from respiratory diseases.

**Table 2:** Clinical characteristics of the study sample, overall and by NUTS statistical region

Characteristic	Italy	Northwestern Italy	Northeastern Italy	Central Italy	Southern Italy	Insular Italy
	(*n* = 1810)	(*n* = 553)	(*n* = 361)	(*n* = 328)	(*n* = 396)	(*n* = 172)
Problems with daily living tasks due to physical or mental impairment						
Yes	217 (12.0%)	45 (8.1%)	69 (19.1%)	38 (11.6%)	54 (13.6%)	11 (6.4%)
No	1593 (88.0%)	508 (91.9%)	292 (80.9%)	290 (88.4%)	342 (86.4%)	161 (93.6%)
BMI ≥30 kg/m^2^						
Yes	317 (17.5%)	77 (13.9%)	64 (17.7%)	66 (20.1%)	70 (17.7%)	40 (23.3%)
No	1493 (82.5%)	476 (86.1%)	297 (82.3%)	262 (79.9%)	326 (82.3%)	132 (76.7%)
Diabetes						
Yes	442 (24.4%)	104 (18.8%)	103 (28.5%)	66 (20.1%)	118 (29.8%)	51 (29.7%)
No	1368 (75.6%)	449 (81.2%)	258 (71.5%)	262 (79.9%)	278 (70.2%)	121 (70.3%)
Pneumopathy						
Yes	229 (12.7%)	59 (10.7%)	34 (9.4%)	50 (15.2%)	61 (15.4%)	25 (14.5%)
No	1581 (87.3%)	494 (89.3%)	327 (90.6%)	278 (84.8%)	335 (84.6%)	147 (85.5%)
Cardiopathy						
Yes	344 (19.0%)	98 (17.7%)	68 (18.8%)	58 (17.7%)	95 (24.0%)	25 (14.5%)
No	1466 (81.0%)	455 (82.3%)	293 (81.2%)	270 (82.3%)	301 (76.0%)	147 (85.5%)

Northwestern Italy includes the regions of Piedmont, Aosta Valley, Lombardy, and Liguria; Northeastern Italy includes the regions of Trentino-South Tyrol, Veneto, Friuli-Venezia Giulia, and Emilia-Romagna; Central Italy includes the regions of Tuscany, Umbria, Marche, and Lazio; Southern Italy includes the regions of Abruzzo, Molise, Campania, Apulia, Basilicata, and Calabria; Insular Italy includes the regions of Sicily and Sardinia

*NUTS* Nomenclature of Territorial Units for Statistics, *BMI* body mass index

### Geographical and class risk stratified uptake

**Fig. 1:**
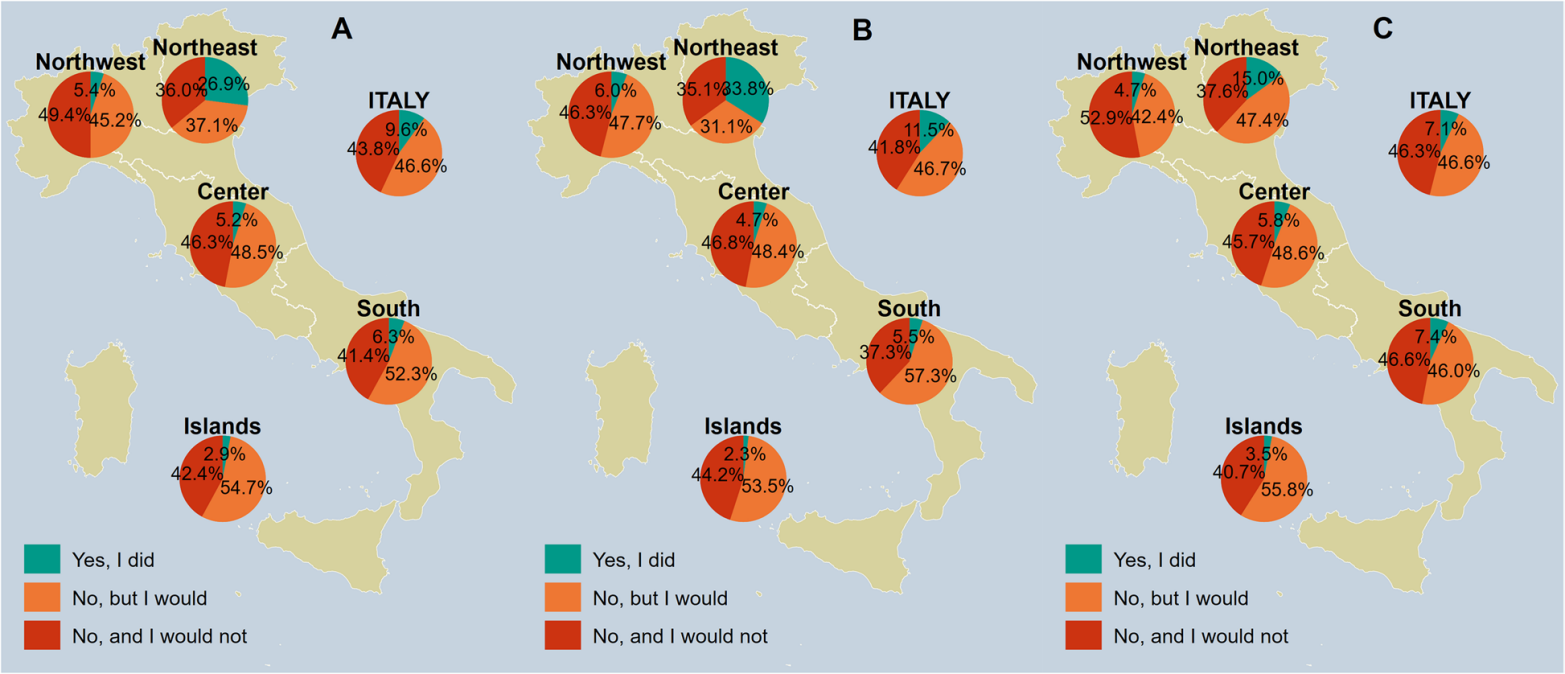
Zoster vaccine uptake by NUTS statistical region, overall (**A**) and in males (**B**) vs. females (**C**); if the answer is no, the respondents are asked whether they would get the vaccine. *Notes:* Females include non-binary people. Northwestern Italy includes the regions of Piedmont, Aosta Valley, Lombardy, and Liguria; Northeastern Italy includes the regions of Trentino-South Tyrol, Veneto, Friuli-Venezia Giulia, and Emilia-Romagna; Central Italy includes the regions of Tuscany, Umbria, Marche, and Lazio; Southern Italy includes the regions of Abruzzo, Molise, Campania, Apulia, Basilicata, and Calabria; Insular Italy includes the regions of Sicily and Sardinia. *NUTS*, Nomenclature of Territorial Units for Statistics

Overall, the reported vaccine uptake in Italy was 9.6%, with a maximum uptake in the Northeast (26.9%) and a minimum in the Islands (2.9%), as illustrated in Fig. 1A. In all regions almost half of those who did not receive the vaccination reported that they would get it.

Figures 1B and 1 C illustrate the vaccine uptake stratified by gender. Males reported being vaccinated in 11.5% of cases, while females in 7.1%. Both males and females reported the highest vaccine uptake in the Northeast (33.8 and 15.0%, respectively) and the lowest in the Islands (2.3 and 3.5%).

**Fig. 2:**
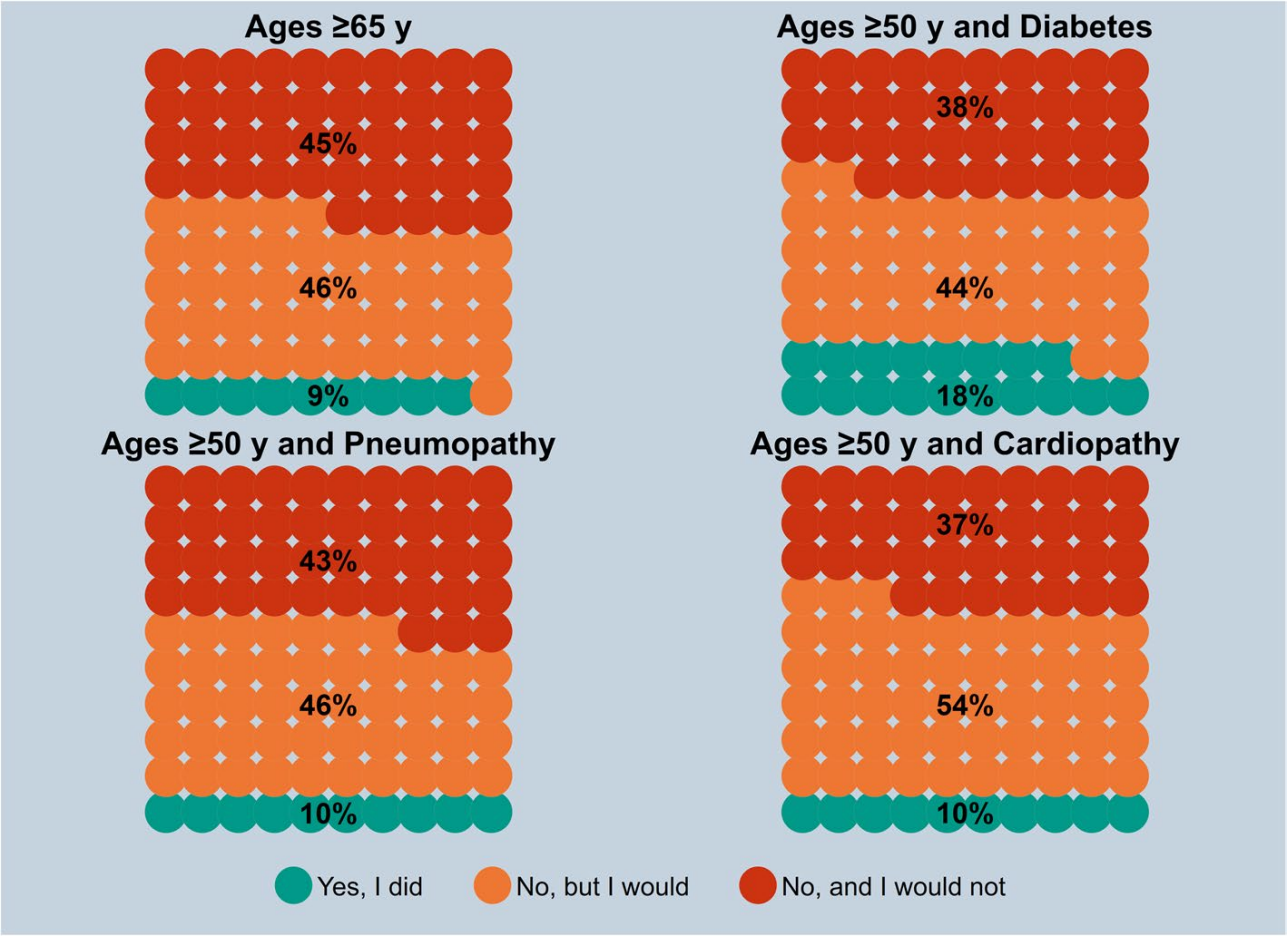
Zoster vaccine uptake by high-risk target group based on age and/or clinical status; if the answer is no, the respondents are asked whether they would get the vaccine

Considering the risk conditions, as shown in Fig. 2, among the respondents ≥65 y.o.,9% reported to having received the vaccination, 46% reported not having received it but they would consider getting it, and 45% reported not having received it and would not get it. In respondents ≥50 y.o. with diabetes, 18% received the vaccination, 44% reported that they would consider getting it and 38% that would not get it. Both 10% of the respondents over 50 y.o. with pneumopathy or cardiopathy reported that they received the vaccination, 46 and 54% respectively reported that they would consider getting the shot.

### Perception and ease of access

As displayed by Supplemental Table 1, our investigation of vaccine-related experiences revealed that most respondents (68.3%) received most of the vaccinations in dedicated hubs; nonetheless, when asked where they would prefer to get vaccinated, the most favourite place to get the vaccine was reported to be the family doctor (36.1%), closely followed by vaccine hubs (34.5%). These results did not vary appreciably considering different regions. Regarding friends and family’s views on vaccinations, most respondents reported favourable opinions, ranging from quite favourable to very favourable in a cumulative percentage of 85.5%.

**Fig. 3:**
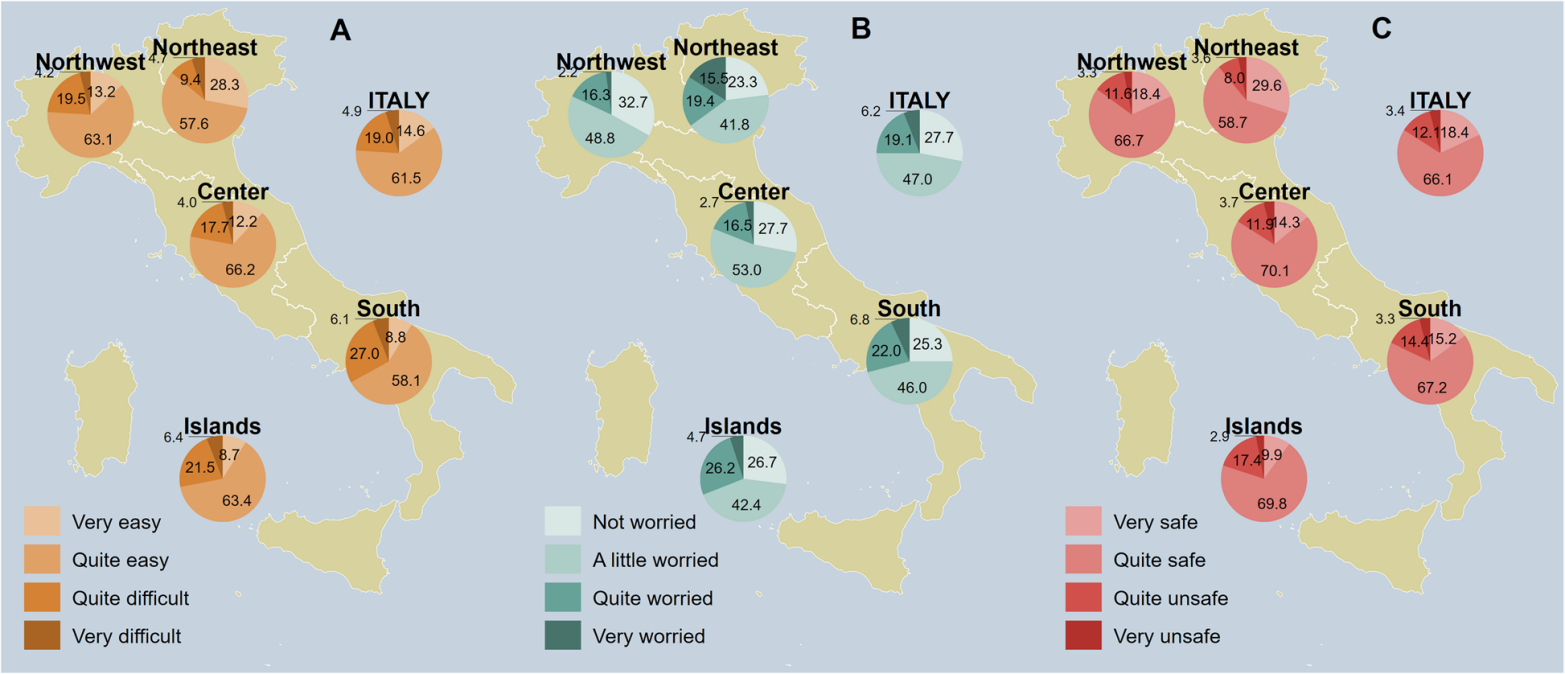
Perception of how easy it is to access healthcare facilities to get a zoster vaccine (**A**), worry about getting sick with shingles (**B**) and perception of the safety of zoster vaccine (**C**), overall and by NUTS statistical region. *Notes:* Northwestern Italy includes the regions of Piedmont, Aosta Valley, Lombardy, and Liguria; Northeastern Italy includes the regions of Trentino-South Tyrol, Veneto, Friuli-Venezia Giulia, and Emilia-Romagna; Central Italy includes the regions of Tuscany, Umbria, Marche, and Lazio; Southern Italy includes the regions of Abruzzo, Molise, Campania, Apulia, Basilicata, and Calabria; Insular Italy includes the regions of Sicily and Sardinia. *NUTS*, Nomenclature of Territorial Units for Statistics

Figure 3A shows responses’ distribution regarding ease of access; overall, the access to vaccination was perceived as very/quite easy by 76.1% of our sample, with the lowest reported in the South (66.9%) and the highest in the Northeast (85.9%). As it comes to worry about the disease, displayed in Fig. 3B, the least worried region was the Northwest with 32.7% of respondents claiming not to be concerned by it. In contrast, Northeast was the most worried region, with 15.5% of respondents reporting to be very worried about the possibility of developing HZ.

Figure 3C displays perception of safety. Most Italians deemed the vaccine against shingles quite safe (66.1%); Islands were the least confident in vaccine safety, with 20.3% of respondents reporting the vaccine to be deemed from quite unsafe to very unsafe. In contrast, Italy’s Northeast was the most confident about vaccine safety, with 29.6% of respondents reporting to consider the vaccine to be very safe.

Data representation of all these matters can be found, stratified by gender and pathologic conditions, in the Supplementary material (S-Fig. A1, A2; S-Fig. B1, B2; S-Fig. C1, C2).

### Multivariable regression analysis

As shown in Table 3, being female, having high school education or less, being unworried about shingles, perceiving shingles vaccine as unsafe, having friends or relatives against vaccination, and being unaware of being a target for shingles vaccination were significantly associated with higher probability of refusing vaccination, while a significant predictor of delay in acceptance was self-reported difficulty in access to healthcare, as well as living in Insular Italy. It was also found that living in Northeastern Italy, living in rural areas, and suffering from diabetes significantly increased the probability of vaccine uptake.

**Table 3:** Results of multivariable multinomial logistic regression analysis: determinants of zoster vaccine uptake and hesitancy (expressed as delay vs. refusal) (*n* = 1810)

	Did get the vaccine			Would get the vaccine			Would not get the vaccine		
Characteristic	Predicted probability	Discrete difference (*Δ*)		Predicted probability	Discrete difference (*Δ*)		Predicted probability	Discrete difference (*Δ*)	
		Estimate	95% CI		Estimate	95% CI		Estimate	95% CI
Gender									
Male	10.4%			48.2%			41.5%		
Female†	8.6%	−1.7	−4.0, 0.6	44.6%	−3.6	−7.6, 0.4	46.8%	5.3*	1.6, 9.0
Age group, y									
< 65	8.0%			51.1%			40.9%		
≥ 65	10.2%	2.2	−0.5, 4.9	45.2%	−5.9*	−11.4, −0.5	44.6%	3.7	−1.4, 8.8
NUTS statistical region									
Northwestern Italy	8.5%			46.3%			45.2%		
Northeastern Italy	15.6%	7.1*	3.3, 10.9	37.2%	−9.2*	−15.0, −3.3	47.3%	2.0	−3.4, 7.5
Central Italy	6.9%	−1.5	−5.1, 2.0	49.9%	3.6	−2.2, 9.5	43.1%	−2.1	−7.2, 3.1
Southern Italy	7.0%	−1.4	−4.8, 1.9	51.4%	5.0	−0.6, 10.7	41.6%	−3.6	−8.6, 1.4
Insular Italy	4.0%	−4.4*	−8.3, −0.6	56.4%	10.1*	2.7, 17.5	39.5%	−5.7	−12.4, 1.0
Degree of urbanisation‡									
City	9.0%			47.7%			43.3%		
Town or suburb	9.0%	0.0	−2.5, 2.4	47.0%	−0.7	−5.0, 3.6	44.0%	0.7	−3.2, 4.6
Rural area	13.0%	4.0*	0.0, 8.0	42.8%	−5.0	−11.3, 1.4	44.2%	0.9	−4.9, 6.7
Educational attainment									
Academic/Post-graduate degree	11.2%			50.4%			38.4%		
High school diploma	9.4%	−1.8	−4.7, 1.0	45.6%	−4.8	−9.6, 0.0	45.0%	6.6*	2.2, 11.1
Less than high school diploma	7.8%	−3.4*	−6.9, 0.0	46.2%	−4.2	−10.5, 2.0	46.0%	7.7*	1.9, 13.5
Diabetes									
No	8.5%			47.5%			44.0%		
Yes	12.6%	4.2*	1.0, 7.3	44.5%	−3.0	−8.3, 2.3	42.9%	−1.1	−6.0, 3.7
Pneumopathy									
No	9.3%			47.0%			43.7%		
Yes	12.4%	3.1	−0.8, 7.1	43.7%	−3.3	−9.7, 3.0	43.9%	0.2	−5.7, 6.0
Cardiopathy									
No	9.2%			45.8%			45.0%		
Yes	11.4%	2.2	−1.1, 5.4	50.1%	4.3	− 1.1, 9.7	38.5%	−6.5*	−11.4, − 1.6
Worry about shingles									
Very/Quite worried	12.4%			70.9%			16.7%		
A little worried	8.6%	−3.8*	−6.8, −0.7	48.8%	−22.1*	−27.1, −17.1	42.6%	25.9*	21.4, 30.4
Not worried	8.6%	−3.8*	−7.2, −0.3	24.1%	−46.7*	−52.3, −41.2	67.2%	50.5*	45.2, 55.8
Perception of vaccine safety									
Very safe	13.6%			61.2%			25.2%		
Quite safe	8.6%	−5.0*	−8.9, −1.0	49.5%	−11.7*	−18.1, −5.3	41.9%	16.7*	11.0, 22.3
Quite/Very unsafe	8.3%	−5.2	−10.9, 0.4	17.8%	−43.5*	−51.7, −35.2	73.9%	48.7*	41.0, 56.4
Dear ones’ views on vaccination in general									
Very favorable	8.9%			55.3%			35.9%		
Favorable	9.4%	0.5	−2.3, 3.3	48.5%	−6.7*	−12.0, − 1.5	42.1%	6.2*	1.3, 11.1
Quite favorable	10.8%	2.0	−1.4, 5.3	38.7%	−16.5*	−22.1, −10.9	50.5%	14.6*	9.4, 19.8
Quite to very unfavorable	12.4%	3.5	−1.3, 8.4	35.8%	−19.5*	−27.1, −11.9	51.8%	15.9*	9.1, 22.8
Awareness of having priority for vaccination									
Yes	20.1%			51.2%			28.7%		
No	5.2%	−14.9*	−19.7, −10.2	40.1%	−11.0*	−18.1, −4.0	54.7%	26.0*	19.5, 32.5
Don’t know	3.4%	−16.7*	−20.6, − 12.9	49.9%	−1.2	−6.4, 3.9	46.7%	18.0*	13.3, 22.7
Perceived ease of access to get the vaccine									
Very easy	13.7%			41.0%			45.3%		
Quite easy	8.3%	−5.4*	−9.2, −1.6	46.8%	5.8	−0.7, 12.2	44.9%	−0.4	−6.4, 5.7
Quite/Very difficult	7.9%	−5.8*	−10.3, − 1.4	52.0%	11.0*	3.5, 18.4	40.1%	−5.2	−12.0, 1.7

*P-value ≤0.05, that is, *Δ* significantly ≠ 0

†Including non-binary persons

‡According to the Eurostat Degree of Urbanization (DEGURBA) classification system

*NUTS* Nomenclature of Territorial Units for Statistics

The analysis of possible interaction effects across covariates revealed that the impact of not being aware of being a target for the vaccination on vaccine refusal was lower when people were quite or very worried about shingles (*aware*: 6.8%; *unaware*: 19.5%; Δ = + 12.7; 95% CI = 6.4 to 18.9) as compared to when people were not worried (aware: 43.2%; unaware: 76.1%; Δ = + 32.9; 95% CI = 21.8 to 44.0) (LR test = 24.2, *p*-value = 0.0022). There was also evidence of a significant interaction (LR = 25.6, p-value = 0.0012) between perceived ease of access and vaccine priority awareness, suggesting that the impact of not being aware on vaccine refusal was lower when access was perceived as *very easy* (*aware*: 29.6%; *not aware*: 49.1%; Δ = + 19.5; 95% CI = 8.2 to 30.9) as compared to *difficult* (*aware*: 17.3%; *not aware*: 45.9%; Δ = + 28.7; 95% CI = 19.3 to 38.1).

## Discussion

To our knowledge, this study represents the first nationwide investigation about shingles vaccine uptake and confidence in Italy, and in the EU region.

In the Italian National Vaccination Plan, a desirable coverage target of 50% was set for 2019 in the target cohorts (those aged ≥65 years and those over 50 with comorbidities) [15]. However, the results obtained from this analysis were markedly below the set target, with an overall uptake of 9.6%. The target was not met even when considering the region with the highest uptake.

Moreover, the analysis of the HZ vaccine uptake reveals notable regional disparities, with Northeastern regions having the highest vaccination rate and Islands the lowest. Divergent perceptions of vaccine safety, ease of access to healthcare services, and awareness of being among the target population, compounded by differing regional healthcare system organisations, contribute to this uneven landscape. Furthermore, sociodemographic factors like male gender, lower educational level, and financial challenges, emerged as significant elements influencing vaccine acceptance, as underscored in the existing literature concerning vaccine confidence [16, 17].

Reported difficulties in accessing the vaccine and low awareness of being part of the target population for free vaccination could partially explain the overall low uptake. The impact of being unaware of belonging to the target population on vaccine refusal was lower when access was perceived as very easy (in Southern Italy and the Islands, access is perceived as much more difficult). However, the present study highlights the positive impact of vaccine eligibility awareness and accessible vaccination services on uptake rates.

In the Northeastern regions, the vaccine uptake is confirmed to be significantly higher, as already shown by thematic maps. This outcome cannot be solely attributed to demographic composition nor to other vaccine-related variables, such as safety and awareness, as these are already incorporated into the model. Instead, it might be explained by the different regional organisations of the Italian National Health System, which sets mandatory and recommended vaccinations nationwide but gives regions the liberty to actively propose vaccinations to target populations.

Considering that different regions have diverse populations and needs, vaccination campaigns should be tailored to their specific requirements. As emphasised by Nicholls et al. [18], the vaccination campaigns should take into account the single vaccine characteristics and should highlight both the risks of the disease and the benefits of vaccination for individuals.

Despite the overall low vaccine uptake, across the country half of those who remain unvaccinated expressed their willingness to receive the vaccine. Although this might seem negative considering that half the unvaccinated population perceived obstacles preventing them from getting vaccinated, it could represent a strategic opportunity from the public health perspective, given that population’s willingness and vaccine confidence are a strong foundation for increasing vaccine uptake [19]. It is remarkable that the sum of those declaring the will to get vaccinated and those who already are, consistently exceeds the national target, and is the foundation for achieving the national vaccination goal.

In conclusion, the current research highlights considerable variances in the uptake of HZ vaccination across Italy’s regions. It is clear that demographic, socioeconomic, and geographic elements bear significant influence on the willingness of individuals and populations to receive the vaccine. These findings stress the pivotal role that knowledge of vaccine eligibility and the availability of vaccination facilities play in incrementing uptake percentages.

By uncovering previously unavailable data on the uptake of HZ vaccination in the Italian context, we hope that these findings, combined with the evidence obtained through our study on perceived vaccination barriers, can inform policy decisions to improve vaccination coverage for a disease as prevalent as HZ.

### Role of the funding source

The funding source had no role in the study design, collection, analysis, and interpretation of data, in the writing of the report; and in the decision to submit the paper.

### Limitations

The limitations of this study are inherent to its design. In fact, being a cross-sectional study, it does not allow any cause-effect interaction between the variables analysed and only provides statistical associations. Secondly, although efforts were made to ensure the representativeness of Italy’s demographics in our sample selection, it is important to note that the recruitment process relied on an online paid survey, potentially attracting individuals seeking additional income, which may have led to an overrepresentation of individuals from lower socioeconomic backgrounds.

Finally, crucial aspects such as household income, religion, and other sensitive social characteristics were not explored in this study, thus potentially compromising the size, statistical power, and representativeness of our sample.

### Supplementary Information

**Additional file 1.** Supplementary material regarding tables and figures. Tables and figures further adding context to the article.

## Data Availability

The datasets generated and/or analysed during the current study are not publicly available due to the continuing analyses on it by the OBVIOUS Board, but are available from the corresponding author on reasonable request.

## Abbreviations

BeSD: Behavioural and Social Drivers
CAWI: Computer-Assisted Web Interviewing
CIs: Confidence Intervals
EU: European Union
HZ: Herpes Zoster
IIA: Independence of Irrelevant Alternatives
LR: Likelihood Ratio
NUTS: Nomenclature of Territorial Units for Statistics
OBVIOUS: Observatory on Vaccine Hesitancy in Italy – Online UniBo Surveys
VZV: Varicella Zoster Virus
WHO: World Health Organization
