# Supplementary material for: Herpes zoster (HZ) vaccine coverage and confidence in Italy: a Nationwide cross-sectional study, the OBVIOUS project
Source: BMC Infect Dis. 2024 Apr 24;24:438. doi: 10.1186/s12879-024-09344-7 (PMC11044443; doi:10.1186/s12879-024-09344-7)
Supplement: Supplementary file 1 — Additional file 1. Supplementary material regarding tables and figures. Tables and figures further adding context to the article. [file 12879_2024_9344_MOESM1_ESM.docx]

**Supplementary**

**Questionnaire**

**1. Gender***

Male

Female

Non binary

**2. Year of birth***

**3. Education level***

1. Elementary/middle school
2. High school
3. University
4. Postgraduate education

**4. What is the postcode of the area you live in*?**

Free field: postcode (e.g. 40126)

**5. Occupation***

1. Student
2. Doctor
3. Other health worker
4. Law enforcement
5. Teacher
6. Employed (other category than above)
7. Unemployed
8. Retired

**6. Who do you live with*?**

1. I live alone
2. I live as a couple
3. I live with my family of origin
4. Other

**7. With the financial resources available to you (from your own or your family's income) can you meet the needs of your current living situation*?**

1. With great difficulty
2. With some difficulty
3. Quite easily
4. Easily

**8. Do you have any children*?**

1. Yes
2. No

**9. What is the sex of your youngest child?**

1. M
2. F

**10. Date of birth of the youngest child:**

**11.** **Who makes decisions about vaccination of your children?**

1. Mainly me
2. Mainly my partner
3. Evenly divided

**12. Were you pregnant at the beginning of the influenza epidemic season (October/November 2021)?**

1. Yes
2. No

**13. Due to a physical/psychological/sensory disability, do you have difficulties completing daily tasks such as going to the doctor or buying groceries?**

1. Yes
2. No

**14. Weight**

**15. Height**

**16. Do you have chronic respiratory diseases (i.e.: severe asthma, bronchopulmonary dysplasia, cystic fibrosis and chronic obstructive pulmonary disease-BPCO)?**

1. Yes
2. No

**17. Do you have any cardiovascular chronic diseases (i.e.: congenital and acquired heart disease)?**

1. Yes
2. No

**18. Are you diabetic?**

1. Yes
2. No

**19. In which of the following facilities did you received most of your vaccinations*?**

1. in a hospital
2. in a pharmacy
3. at your family doctor's
4. in a vaccination hub
5. at home
6. at work

**20. If you could choose, in which facility/place would you prefer to receive a vaccination*?**

1. in a hospital
2. in a pharmacy
3. at your family doctor’s
4. in a vaccination hub
5. at home
6. at work

**21. How do your family and friends feel about vaccinations**?**

1. Very unfavorable
2. Unfavorable
3. Quite unfavorable
4. Quite favorable
5. Favorable
6. Very favorable

**22. Did you receive the vaccination for Herpes Zoster (commonly named shingles)?**

1. Yes
2. No
3. I don't know what it is → close section

**23.** **Would you get the vaccination for Herpes Zoster?**

1. Yes
2. No

**24. How worried are you about getting Herpes Zoster?**

1. Not worried
2. A little worried
3. Quite worried
4. Very worried

**25. How safe do you think the vaccine for Herpes Zoster is?**

1. Very safe
2. Quite safe
3. Quite unsafe
4. Very unsafe

**26. Do you think you are entitled to receive a free Herpes Zoster vaccination?**

1. Yes
2. No

**27. How easy do you think it is for you to access facilities to get the Herpes Zoster vaccination?**

1. Very easy
2. Quite easy
3. Quite difficult
4. Very difficult

**FIGURES**

**S-Figure A1.** Perception of how easy it is to access healthcare facilities to get a zoster vaccine in males vs. females (*n* = 1810), overall and by NUTS statistical region (%).


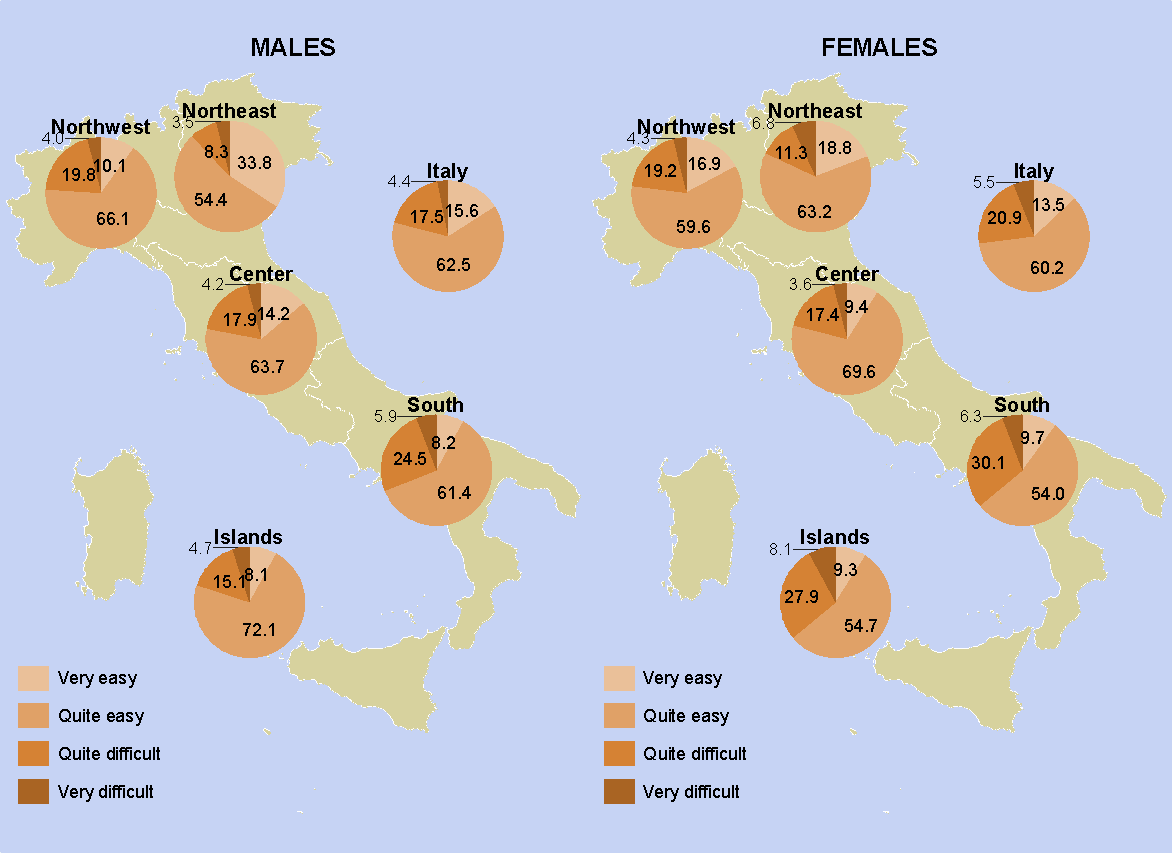


*Notes:* Females include non-binary persons. Northwestern Italy includes the regions of Piedmont, Aosta Valley, Lombardy, and Liguria; Northeastern Italy includes the regions of Trentino-South Tyrol, Veneto, Friuli-Venezia Giulia, and Emilia-Romagna; Central Italy includes the regions of Tuscany, Umbria, Marche, and Lazio; Southern Italy includes the regions of Abruzzo, Molise, Campania, Apulia, Basilicata, and Calabria; Insular Italy includes the regions of Sicily and Sardinia. *NUTS*, Nomenclature of Territorial Units for Statistics.

**S-Figure A2.** Perception of how easy it is to access healthcare facilities to get a zoster vaccine, by high-risk target group based on age and/or clinical status.

*
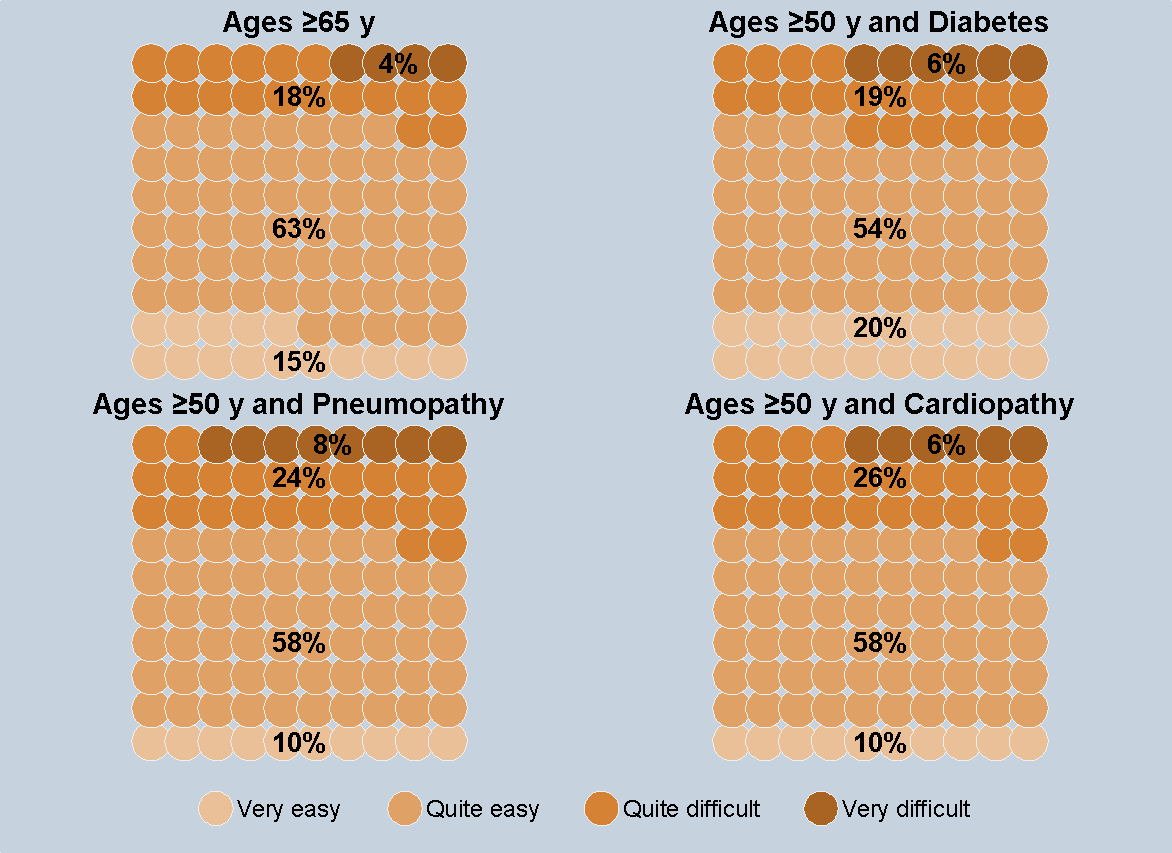
*

**S-Figure B1.** Worry about getting sick with shingles in males vs. females (*n* = 1810), overall and by NUTS statistical region.


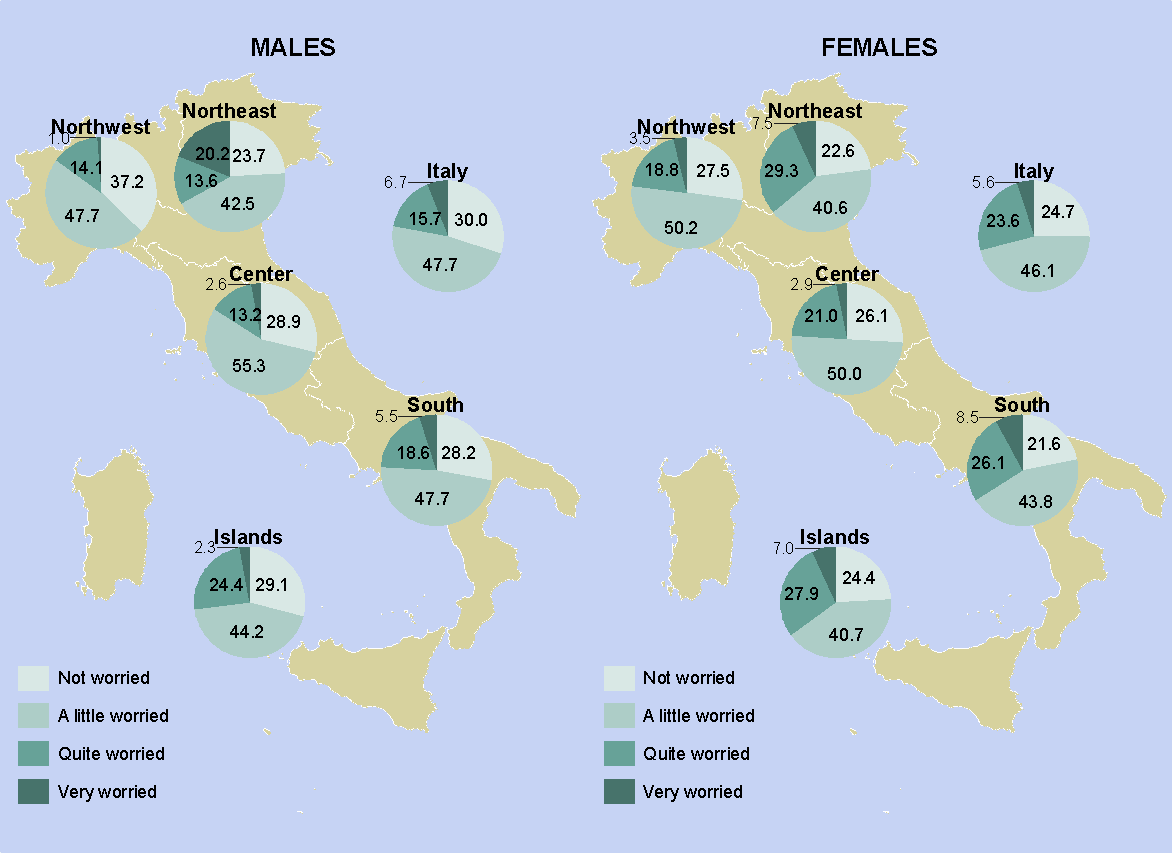


*Notes:* Northwestern Italy includes the regions of Piedmont, Aosta Valley, Lombardy, and Liguria; Northeastern Italy includes the regions of Trentino-South Tyrol, Veneto, Friuli-Venezia Giulia, and Emilia-Romagna; Central Italy includes the regions of Tuscany, Umbria, Marche, and Lazio; Southern Italy includes the regions of Abruzzo, Molise, Campania, Apulia, Basilicata, and Calabria; Insular Italy includes the regions of Sicily and Sardinia. *NUTS*, Nomenclature of Territorial Units for Statistics.

**S-Figure B2.** Worry about getting sick with shingles, by high-risk target group based on age and/or clinical status.

**
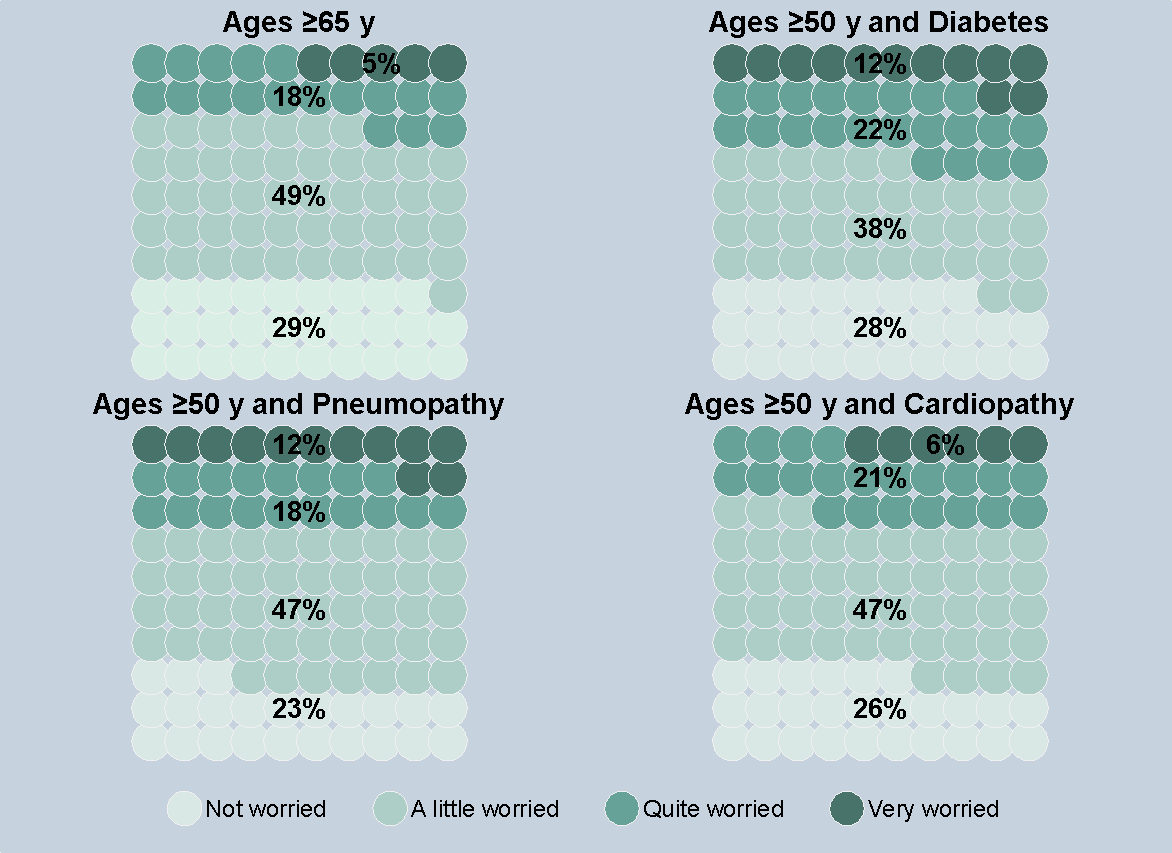
**

**S-Figure C1.** Perception of the safety of zoster vaccine in males vs. females (*n* = 1810), overall and by NUTS statistical region (%).


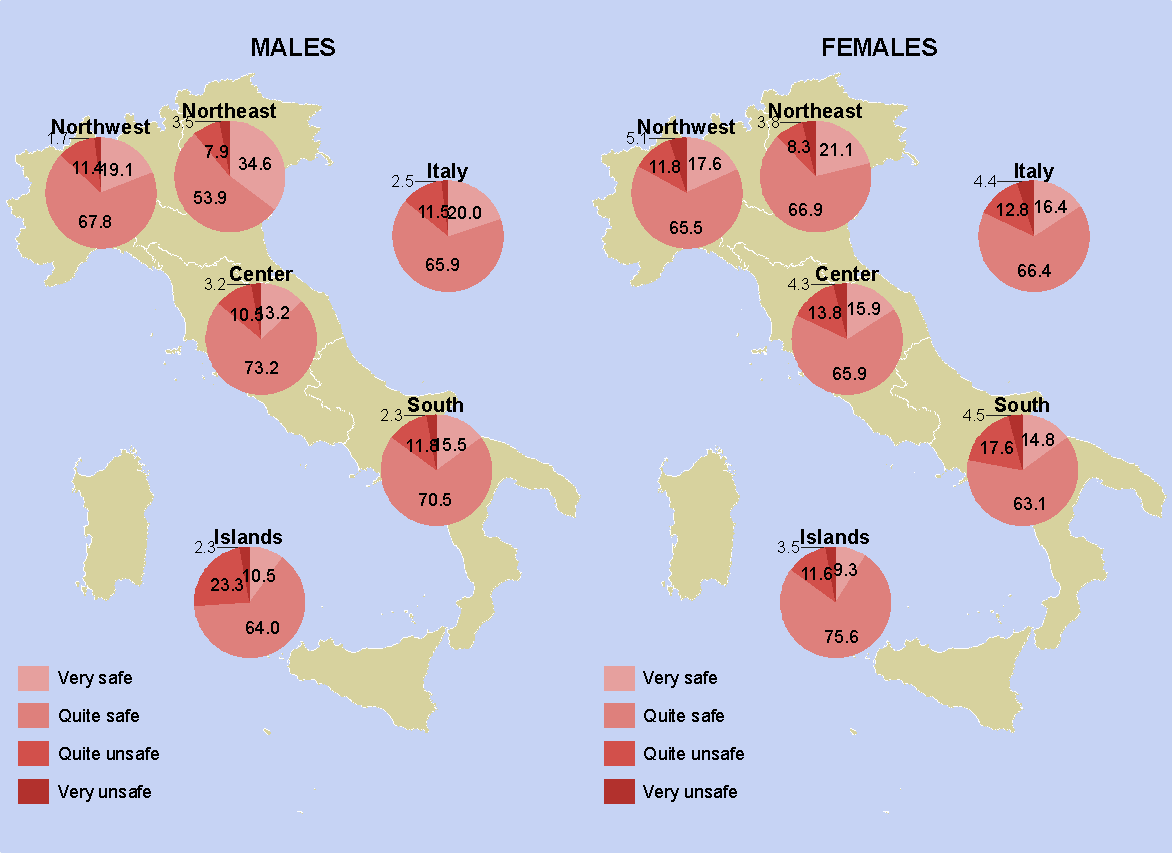


*Notes:* Females include non-binary persons. Northwestern Italy includes the regions of Piedmont, Aosta Valley, Lombardy, and Liguria; Northeastern Italy includes the regions of Trentino-South Tyrol, Veneto, Friuli-Venezia Giulia, and Emilia-Romagna; Central Italy includes the regions of Tuscany, Umbria, Marche, and Lazio; Southern Italy includes the regions of Abruzzo, Molise, Campania, Apulia, Basilicata, and Calabria; Insular Italy includes the regions of Sicily and Sardinia. *NUTS*, Nomenclature of Territorial Units for Statistics.

**S-Figure C2** Perception of the safety of zoster vaccines, by high-risk target group based on age and/or clinical status.

*
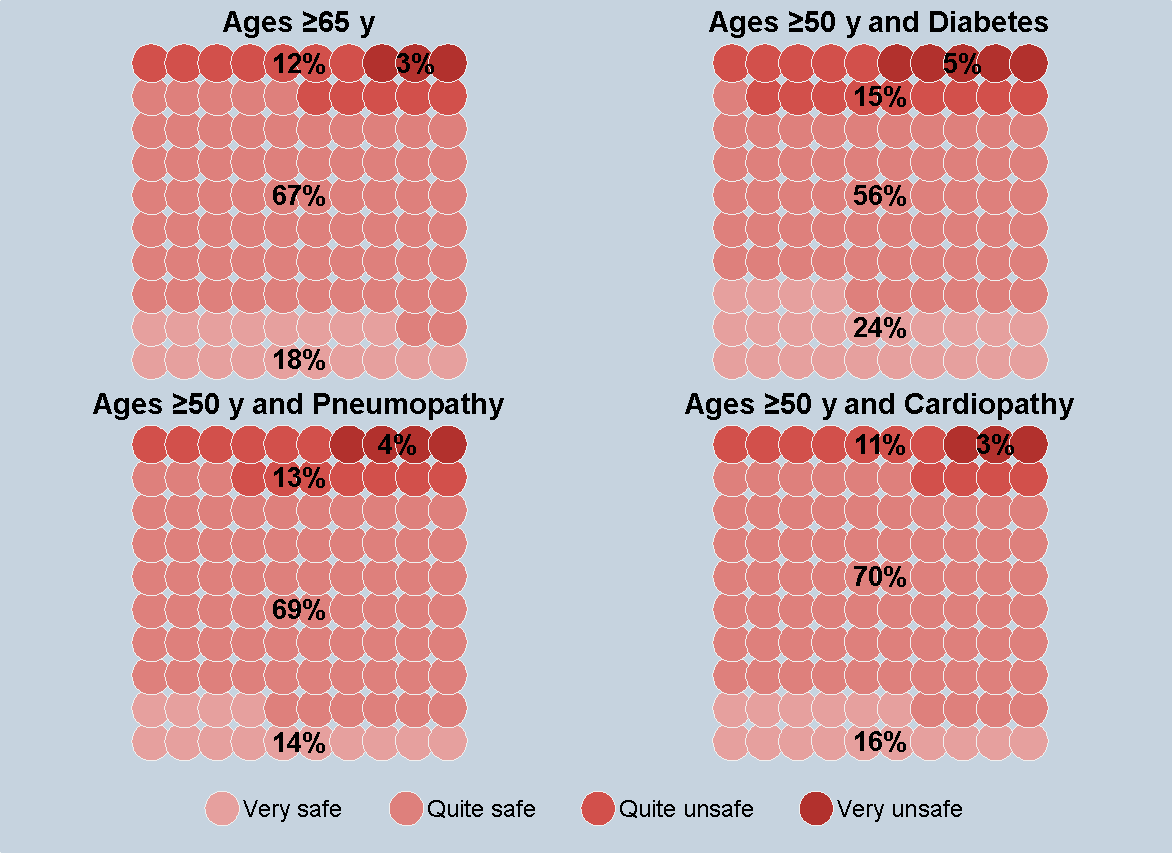
*

**TABLES**

**S-Table 1.** General information about vaccines, overall and by NUTS statistical region.

| Characteristic | Italy | Northwestern Italy | Northeastern Italy | Central Italy | Southern Italy | Insular Italy |
| --- | --- | --- | --- | --- | --- | --- |
|  | (*n* = 1810) | (*n* = 553) | (*n* = 361) | (*n* = 328) | (*n* = 396) | (*n* = 172) |
| Place where you prevalently got vaccines |  |  |  |  |  |  |
| Vaccine Hub | 1237 (68.3%) | 418 (75.6%) | 225 (62.3%) | 205 (62.5%) | 273 (68.9%) | 116 (67.4%) |
| Hospital | 255 (14.1%) | 65 (11.8%) | 40 (11.1%) | 64 (19.5%) | 55 (13.9%) | 31 (18.0%) |
| Family doctor | 254 (14.0%) | 50 (9.0%) | 81 (22.4%) | 47 (14.3%) | 54 (13.6%) | 22 (12.8%) |
| Home | 25 (1.4%) | 7 (1.3%) | 5 (1.4%) | 5 (1.5%) | 7 (1.8%) | 1 (0.6%) |
| Workplace | 20 (1.1%) | 5 (0.9%) | 10 (2.8%) | 2 (0.6%) | 1 (0.3%) | 2 (1.2%) |
| Pharmacy | 19 (1.0%) | 8 (1.4%) | 0 (0.0%) | 5 (1.5%) | 6 (1.5%) | 0 (0.0%) |
| Favorite place to get vaccines |  |  |  |  |  |  |
| Family doctor | 654 (36.1%) | 166 (30.0%) | 160 (44.3%) | 113 (34.5%) | 143 (36.1%) | 72 (41.9%) |
| Vaccine Hub | 625 (34.5%) | 219 (39.6%) | 111 (30.7%) | 106 (32.3%) | 138 (34.8%) | 51 (29.7%) |
| Hospital | 273 (15.1%) | 84 (15.2%) | 42 (11.6%) | 65 (19.8%) | 53 (13.4%) | 29 (16.9%) |
| Home | 121 (6.7%) | 31 (5.6%) | 21 (5.8%) | 21 (6.4%) | 33 (8.3%) | 15 (8.7%) |
| Pharmacy | 114 (6.3%) | 49 (8.9%) | 20 (5.5%) | 18 (5.5%) | 23 (5.8%) | 4 (2.3%) |
| Workplace | 23 (1.3%) | 4 (0.7%) | 7 (1.9%) | 5 (1.5%) | 6 (1.5%) | 1 (0.6%) |
| Friends and family’s views on vaccination |  |  |  |  |  |  |
| Very unfavorable | 84 (4.6%) | 28 (5.1%) | 17 (4.7%) | 15 (4.6%) | 20 (5.1%) | 4 (2.3%) |
| Unfavorable | 52 (2.9%) | 12 (2.2%) | 14 (3.9%) | 9 (2.7%) | 10 (2.5%) | 7 (4.1%) |
| Quite unfavorable | 125 (6.9%) | 38 (6.9%) | 21 (5.8%) | 30 (9.1%) | 22 (5.6%) | 14 (8.1%) |
| Quite favorable | 471 (26.0%) | 122 (22.1%) | 71 (19.7%) | 86 (26.2%) | 125 (31.6%) | 67 (39.0%) |
| Favorable | 518 (28.6%) | 168 (30.4%) | 100 (27.7%) | 91 (27.7%) | 114 (28.8%) | 45 (26.2%) |
| Very favorable | 560 (30.9%) | 185 (33.5%) | 138 (38.2%) | 97 (29.6%) | 105 (26.5%) | 35 (20.3%) |

*Notes:* Northwestern Italy includes the regions of Piedmont, Aosta Valley, Lombardy, and Liguria; Northeastern Italy includes the regions of Trentino-South Tyrol, Veneto, Friuli-Venezia Giulia, and Emilia-Romagna; Central Italy includes the regions of Tuscany, Umbria, Marche, and Lazio; Southern Italy includes the regions of Abruzzo, Molise, Campania, Apulia, Basilicata, and Calabria; Insular Italy includes the regions of Sicily and Sardinia. *NUTS*, Nomenclature of Territorial Units for Statistics.

**S-Table 2.** Zoster vaccine uptake by region of residence; if the answer is no, the respondents are asked whether or not they would get the vaccine.

| Region | All | Yes, I did | No, but I would | No, and I would |
| --- | --- | --- | --- | --- |
|  |  |  |  | not |
| Northwestern Italy |  |  |  |  |
| Piedmont | 171 (100%) | 11 (6%) | 79 (46%) | 81 (47%) |
| Aosta Valley | 4 (100%) | 0 (0%) | 1 (25%) | 3 (75%) |
| Lombardy | 318 (100%) | 11 (3%) | 148 (47%) | 159 (50%) |
| Liguria | 60 (100%) | 8 (13%) | 22 (37%) | 30 (50%) |
| Northeastern Italy |  |  |  |  |
| Trentino-South Tyrol | 21 (100%) | 9 (43%) | 5 (24%) | 7 (33%) |
| Veneto | 147 (100%) | 64 (44%) | 41 (28%) | 42 (29%) |
| Friuli-Venezia Giulia | 57 (100%) | 4 (7%) | 23 (40%) | 30 (53%) |
| Emilia-Romagna | 136 (100%) | 20 (15%) | 65 (48%) | 51 (38%) |
| Central Italy |  |  |  |  |
| Tuscany | 93 (100%) | 4 (4%) | 50 (54%) | 39 (42%) |
| Umbria | 25 (100%) | 1 (4%) | 12 (48%) | 12 (48%) |
| Marche | 57 (100%) | 4 (7%) | 33 (58%) | 20 (35%) |
| Lazio | 153 (100%) | 8 (5%) | 64 (42%) | 81 (53%) |
| Southern Italy |  |  |  |  |
| Abruzzo | 44 (100%) | 3 (7%) | 22 (50%) | 19 (43%) |
| Molise | 6 (100%) | 0 (0%) | 4 (67%) | 2 (33%) |
| Campania | 172 (100%) | 10 (6%) | 88 (51%) | 74 (43%) |
| Apulia | 104 (100%) | 5 (5%) | 55 (53%) | 44 (42%) |
| Basilicata | 17 (100%) | 1 (6%) | 15 (88%) | 1 (6%) |
| Calabria | 53 (100%) | 6 (11%) | 23 (43%) | 24 (45%) |
| Insular Italy |  |  |  |  |
| Sicily | 112 (100%) | 5 (4%) | 58 (52%) | 49 (44%) |
| Sardinia | 60 (100%) | 0 (0%) | 36 (60%) | 24 (40%) |

**S-Table 3A.** Perception of how easy it is to access healthcare facilities to get a zoster vaccine, by region of residence.

| Region | All | Very easy | Quite easy | Quite difficult | Very difficult |
| --- | --- | --- | --- | --- | --- |
| Northwestern Italy |  |  |  |  |  |
| Piedmont | 171 (100%) | 36 (21%) | 95 (56%) | 33 (19%) | 7 (4%) |
| Aosta Valley | 4 (100%) | 0 (0%) | 3 (75%) | 1 (25%) | 0 (0%) |
| Lombardy | 318 (100%) | 25 (8%) | 218 (69%) | 61 (19%) | 14 (4%) |
| Liguria | 60 (100%) | 12 (20%) | 33 (55%) | 13 (22%) | 2 (3%) |
| Northeastern Italy |  |  |  |  |  |
| Trentino-South Tyrol | 21 (100%) | 9 (43%) | 9 (43%) | 3 (14%) | 0 (0%) |
| Veneto | 147 (100%) | 50 (34%) | 77 (52%) | 15 (10%) | 5 (3%) |
| Friuli-Venezia Giulia | 57 (100%) | 13 (23%) | 36 (63%) | 7 (12%) | 1 (2%) |
| Emilia-Romagna | 136 (100%) | 30 (22%) | 86 (63%) | 9 (7%) | 11 (8%) |
| Central Italy |  |  |  |  |  |
| Tuscany | 93 (100%) | 8 (9%) | 69 (74%) | 12 (13%) | 4 (4%) |
| Umbria | 25 (100%) | 6 (24%) | 15 (60%) | 4 (16%) | 0 (0%) |
| Marche | 57 (100%) | 5 (9%) | 42 (74%) | 9 (16%) | 1 (2%) |
| Lazio | 153 (100%) | 21 (14%) | 91 (59%) | 33 (22%) | 8 (5%) |
| Southern Italy |  |  |  |  |  |
| Abruzzo | 44 (100%) | 3 (7%) | 28 (64%) | 11 (25%) | 2 (5%) |
| Molise | 6 (100%) | 0 (0%) | 5 (83%) | 1 (17%) | 0 (0%) |
| Campania | 172 (100%) | 14 (8%) | 97 (56%) | 51 (30%) | 10 (6%) |
| Apulia | 104 (100%) | 8 (8%) | 63 (61%) | 29 (28%) | 4 (4%) |
| Basilicata | 17 (100%) | 3 (18%) | 11 (65%) | 1 (6%) | 2 (12%) |
| Calabria | 53 (100%) | 7 (13%) | 26 (49%) | 14 (26%) | 6 (11%) |
| Insular Italy |  |  |  |  |  |
| Sicily | 112 (100%) | 10 (9%) | 72 (64%) | 24 (21%) | 6 (5%) |
| Sardinia | 60 (100%) | 5 (8%) | 37 (62%) | 13 (22%) | 5 (8%) |

**S-Table 3B.** Worry about getting sick with shingles by region of residence.

| Region | All | Not worried | A little worried | Quite worried | Very worried |
| --- | --- | --- | --- | --- | --- |
| Northwestern Italy |  |  |  |  |  |
| Piedmont | 171 (100%) | 53 (31%) | 73 (43%) | 40 (23%) | 5 (3%) |
| Aosta Valley | 4 (100%) | 2 (50%) | 2 (50%) | 0 (0%) | 0 (0%) |
| Lombardy | 318 (100%) | 103 (32%) | 164 (52%) | 47 (15%) | 4 (1%) |
| Liguria | 60 (100%) | 23 (38%) | 31 (52%) | 3 (5%) | 3 (5%) |
| Northeastern Italy |  |  |  |  |  |
| Trentino-South Tyrol | 21 (100%) | 3 (14%) | 9 (43%) | 4 (19%) | 5 (24%) |
| Veneto | 147 (100%) | 28 (19%) | 50 (34%) | 28 (19%) | 41 (28%) |
| Friuli-Venezia Giulia | 57 (100%) | 22 (39%) | 28 (49%) | 5 (9%) | 2 (4%) |
| Emilia-Romagna | 136 (100%) | 31 (23%) | 64 (47%) | 33 (24%) | 8 (6%) |
| Central Italy |  |  |  |  |  |
| Tuscany | 93 (100%) | 21 (23%) | 57 (61%) | 15 (16%) | 0 (0%) |
| Umbria | 25 (100%) | 11 (44%) | 8 (32%) | 5 (20%) | 1 (4%) |
| Marche | 57 (100%) | 9 (16%) | 30 (53%) | 15 (26%) | 3 (5%) |
| Lazio | 153 (100%) | 50 (33%) | 79 (52%) | 19 (12%) | 5 (3%) |
| Southern Italy |  |  |  |  |  |
| Abruzzo | 44 (100%) | 18 (41%) | 18 (41%) | 5 (11%) | 3 (7%) |
| Molise | 6 (100%) | 1 (17%) | 2 (33%) | 2 (33%) | 1 (17%) |
| Campania | 172 (100%) | 37 (22%) | 92 (53%) | 39 (23%) | 4 (2%) |
| Apulia | 104 (100%) | 30 (29%) | 45 (43%) | 23 (22%) | 6 (6%) |
| Basilicata | 17 (100%) | 3 (18%) | 2 (12%) | 5 (29%) | 7 (41%) |
| Calabria | 53 (100%) | 11 (21%) | 23 (43%) | 13 (25%) | 6 (11%) |
| Insular Italy |  |  |  |  |  |
| Sicily | 112 (100%) | 30 (27%) | 46 (41%) | 32 (29%) | 4 (4%) |
| Sardinia | 60 (100%) | 16 (27%) | 27 (45%) | 13 (22%) | 4 (7%) |

**S-Table 3C.** Perception of the safety of zoster vaccines by region of residence

| Region | All | Very safe | Quite safe | Quite unsafe | Very unsafe |
| --- | --- | --- | --- | --- | --- |
| Northwestern Italy |  |  |  |  |  |
| Piedmont | 171 (100%) | 41 (24%) | 105 (61%) | 19 (11%) | 6 (4%) |
| Aosta Valley | 4 (100%) | 0 (0%) | 3 (75%) | 1 (25%) | 0 (0%) |
| Lombardy | 318 (100%) | 48 (15%) | 225 (71%) | 35 (11%) | 10 (3%) |
| Liguria | 60 (100%) | 13 (22%) | 36 (60%) | 9 (15%) | 2 (3%) |
| Northeastern Italy |  |  |  |  |  |
| Trentino-South Tyrol | 21 (100%) | 6 (29%) | 15 (71%) | 0 (0%) | 0 (0%) |
| Veneto | 147 (100%) | 58 (39%) | 75 (51%) | 8 (5%) | 6 (4%) |
| Friuli-Venezia Giulia | 57 (100%) | 15 (26%) | 31 (54%) | 10 (18%) | 1 (2%) |
| Emilia-Romagna | 136 (100%) | 28 (21%) | 91 (67%) | 11 (8%) | 6 (4%) |
| Central Italy |  |  |  |  |  |
| Tuscany | 93 (100%) | 12 (13%) | 71 (76%) | 7 (8%) | 3 (3%) |
| Umbria | 25 (100%) | 4 (16%) | 17 (68%) | 4 (16%) | 0 (0%) |
| Marche | 57 (100%) | 10 (18%) | 37 (65%) | 10 (18%) | 0 (0%) |
| Lazio | 153 (100%) | 21 (14%) | 105 (69%) | 18 (12%) | 9 (6%) |
| Southern Italy |  |  |  |  |  |
| Abruzzo | 44 (100%) | 5 (11%) | 34 (77%) | 3 (7%) | 2 (5%) |
| Molise | 6 (100%) | 0 (0%) | 5 (83%) | 1 (17%) | 0 (0%) |
| Campania | 172 (100%) | 22 (13%) | 121 (70%) | 24 (14%) | 5 (3%) |
| Apulia | 104 (100%) | 14 (13%) | 68 (65%) | 20 (19%) | 2 (2%) |
| Basilicata | 17 (100%) | 7 (41%) | 8 (47%) | 1 (6%) | 1 (6%) |
| Calabria | 53 (100%) | 12 (23%) | 30 (57%) | 8 (15%) | 3 (6%) |
| Insular Italy |  |  |  |  |  |
| Sicily | 112 (100%) | 8 (7%) | 83 (74%) | 19 (17%) | 2 (2%) |
| Sardinia | 60 (100%) | 9 (15%) | 37 (62%) | 11 (18%) | 3 (5%) |

**S-Table 4.** Zoster vaccine uptake by gender and high-risk target group based on age and/or clinical status; if the answer is no, the respondents are asked whether or not they would get the vaccine.

|  | All | Yes, I did | No, but I would | No, and I would |
| --- | --- | --- | --- | --- |
|  |  |  |  | not |
| Ages ≥60 y |  |  |  |  |
| All | 1382 (100.0%) | 129 (9.3%) | 631 (45.7%) | 622 (45.0%) |
| Males | 789 (100.0%) | 84 (10.6%) | 363 (46.0%) | 342 (43.3%) |
| Females | 593 (100.0%) | 45 (7.6%) | 268 (45.2%) | 280 (47.2%) |
| Ages ≥50 y and diabetes |  |  |  |  |
| All | 442 (100.0%) | 79 (17.9%) | 193 (43.7%) | 170 (38.5%) |
| Males | 305 (100.0%) | 69 (22.6%) | 125 (41.0%) | 111 (36.4%) |
| Females | 137 (100.0%) | 10 (7.3%) | 68 (49.6%) | 59 (43.1%) |
| Ages ≥50 y and pneumopathy |  |  |  |  |
| All | 229 (100.0%) | 24 (10.5%) | 106 (46.3%) | 99 (43.2%) |
| Males | 106 (100.0%) | 13 (12.3%) | 52 (49.1%) | 41 (38.7%) |
| Females | 123 (100.0%) | 11 (8.9%) | 54 (43.9%) | 58 (47.2%) |
| Ages ≥50 y and cardiopathy |  |  |  |  |
| All | 344 (100.0%) | 33 (9.6%) | 185 (53.8%) | 126 (36.6%) |
| Males | 218 (100.0%) | 26 (11.9%) | 120 (55.0%) | 72 (33.0%) |
| Females | 126 (100.0%) | 7 (5.6%) | 65 (51.6%) | 54 (42.9%) |

*Notes:* Females include non-binary persons.

**S-Table 5.** Worry about getting sick with shingles, by gender and high-risk target group based on age and/or clinical status.

|  | All | Not worried | A little worried | Quite worried | Very worried |
| --- | --- | --- | --- | --- | --- |
| Ages ≥60 y |  |  |  |  |  |
| All | 1382 (100.0%) | 396 (28.7%) | 671 (48.6%) | 249 (18.0%) | 66 (4.8%) |
| Males | 789 (100.0%) | 245 (31.1%) | 393 (49.8%) | 113 (14.3%) | 38 (4.8%) |
| Females | 593 (100.0%) | 151 (25.5%) | 278 (46.9%) | 136 (22.9%) | 28 (4.7%) |
| Ages ≥50 y and diabetes |  |  |  |  |  |
| All | 442 (100.0%) | 123 (27.8%) | 168 (38.0%) | 96 (21.7%) | 55 (12.4%) |
| Males | 305 (100.0%) | 89 (29.2%) | 119 (39.0%) | 50 (16.4%) | 47 (15.4%) |
| Females | 137 (100.0%) | 34 (24.8%) | 49 (35.8%) | 46 (33.6%) | 8 (5.8%) |
| Ages ≥50 y and pneumopathy |  |  |  |  |  |
| All | 229 (100.0%) | 52 (22.7%) | 108 (47.2%) | 42 (18.3%) | 27 (11.8%) |
| Males | 106 (100.0%) | 26 (24.5%) | 55 (51.9%) | 13 (12.3%) | 12 (11.3%) |
| Females | 123 (100.0%) | 26 (21.1%) | 53 (43.1%) | 29 (23.6%) | 15 (12.2%) |
| Ages ≥50 y and cardiopathy |  |  |  |  |  |
| All | 344 (100.0%) | 90 (26.2%) | 161 (46.8%) | 72 (20.9%) | 21 (6.1%) |
| Males | 218 (100.0%) | 55 (25.2%) | 108 (49.5%) | 39 (17.9%) | 16 (7.3%) |
| Females | 126 (100.0%) | 35 (27.8%) | 53 (42.1%) | 33 (26.2%) | 5 (4.0%) |

*Notes:* Females include non-binary persons.

**S-Table 6.** Perception of the safety of zoster vaccines, by gender and high-risk target group based on age and/or clinical status.

|  | All | Very safe | Quite safe | Quite unsafe | Very unsafe |
| --- | --- | --- | --- | --- | --- |
| Ages ≥60 y |  |  |  |  |  |
| All | 1382 (100.0%) | 254 (18.4%) | 920 (66.6%) | 163 (11.8%) | 45 (3.3%) |
| Males | 789 (100.0%) | 154 (19.5%) | 526 (66.7%) | 89 (11.3%) | 20 (2.5%) |
| Females | 593 (100.0%) | 100 (16.9%) | 394 (66.4%) | 74 (12.5%) | 25 (4.2%) |
| Ages ≥50 y and diabetes |  |  |  |  |  |
| All | 442 (100.0%) | 107 (24.2%) | 249 (56.3%) | 66 (14.9%) | 20 (4.5%) |
| Males | 305 (100.0%) | 85 (27.9%) | 165 (54.1%) | 43 (14.1%) | 12 (3.9%) |
| Females | 137 (100.0%) | 22 (16.1%) | 84 (61.3%) | 23 (16.8%) | 8 (5.8%) |
| Ages ≥50 y and pneumopathy |  |  |  |  |  |
| All | 229 (100.0%) | 31 (13.5%) | 159 (69.4%) | 29 (12.7%) | 10 (4.4%) |
| Males | 106 (100.0%) | 15 (14.2%) | 74 (69.8%) | 14 (13.2%) | 3 (2.8%) |
| Females | 123 (100.0%) | 16 (13.0%) | 85 (69.1%) | 15 (12.2%) | 7 (5.7%) |
| Ages ≥50 y and cardiopathy |  |  |  |  |  |
| All | 344 (100.0%) | 55 (16.0%) | 240 (69.8%) | 38 (11.0%) | 11 (3.2%) |
| Males | 218 (100.0%) | 34 (15.6%) | 160 (73.4%) | 22 (10.1%) | 2 (0.9%) |
| Females | 126 (100.0%) | 21 (16.7%) | 80 (63.5%) | 16 (12.7%) | 9 (7.1%) |

*Notes:* Females include non-binary persons.

**S-Table 7.** Perception of how easy it is to access healthcare facilities to get a zoster vaccine, by gender and high-risk target group based on age and/or clinical status.

|  | All | Very easy | Quite easy | Quite difficult | Very difficult |
| --- | --- | --- | --- | --- | --- |
| Ages ≥60 y |  |  |  |  |  |
| All | 1382 (100.0%) | 209 (15.1%) | 865 (62.6%) | 254 (18.4%) | 54 (3.9%) |
| Males | 789 (100.0%) | 120 (15.2%) | 504 (63.9%) | 137 (17.4%) | 28 (3.5%) |
| Females | 593 (100.0%) | 89 (15.0%) | 361 (60.9%) | 117 (19.7%) | 26 (4.4%) |
| Ages ≥50 y and diabetes |  |  |  |  |  |
| All | 442 (100.0%) | 90 (20.4%) | 239 (54.1%) | 85 (19.2%) | 28 (6.3%) |
| Males | 305 (100.0%) | 73 (23.9%) | 164 (53.8%) | 52 (17.0%) | 16 (5.2%) |
| Females | 137 (100.0%) | 17 (12.4%) | 75 (54.7%) | 33 (24.1%) | 12 (8.8%) |
| Ages ≥50 y and pneumopathy |  |  |  |  |  |
| All | 229 (100.0%) | 24 (10.5%) | 132 (57.6%) | 55 (24.0%) | 18 (7.9%) |
| Males | 106 (100.0%) | 15 (14.2%) | 60 (56.6%) | 24 (22.6%) | 7 (6.6%) |
| Females | 123 (100.0%) | 9 (7.3%) | 72 (58.5%) | 31 (25.2%) | 11 (8.9%) |
| Ages ≥50 y and cardiopathy |  |  |  |  |  |
| All | 344 (100.0%) | 35 (10.2%) | 200 (58.1%) | 90 (26.2%) | 19 (5.5%) |
| Males | 218 (100.0%) | 25 (11.5%) | 131 (60.1%) | 51 (23.4%) | 11 (5.0%) |
| Females | 126 (100.0%) | 10 (7.9%) | 69 (54.8%) | 39 (31.0%) | 8 (6.3%) |

*Notes:* Females include non-binary persons.
